# From Hunger to Hospitality: Food, Memory, and Collective Identity in Mohammed Dib’s *La Grande Maison* and Diana Abu-Jaber’s *Crescent*

**DOI:** 10.12688/f1000research.184574.1

**Published:** 2026-09-07

**Authors:** Waffa NOUARI, Amr Mohamed, Mohammed Adel, Hanene Lahiani, Ahmad M. Al Mahamed

**Affiliations:** 1 Arab Open University Oman, Muscat, Muscat Governorate, Oman; 2 North Private College of Nursing, Arar, Saudi Arabia; 3 United Arab Emirates University, Al Ain, Abu Dhabi, United Arab Emirates; 4 Al Ain University College of Education Humanities and Social Sciences, Al Ain, Abu Dhabi, United Arab Emirates; 5 Abu Dhabi University College of Art and Science, Abu Dhabi, Abu Dhabi, United Arab Emirates

**Keywords:** Algerian colonial literature; collective identity; comparative literature; diasporic narratives; food-memory theory; memory

## Abstract

**Background:**

This study explored food memory in Mohammed Dib’s *La Grande Maison* and Diana Abu-Jaber’s *Crescent.* Recent scholarship conceptualizes food as a symbolic and cultural medium that shapes memory, identity, exile, trauma, and belonging rather than merely satisfying physical needs. The analysis reveals how, in both novels, food serves as a medium for communicating and representing historical experience and collective identity.

**Methods:**

Methodologically, the paper uses a qualitative comparative literary approach, comprising close reading of selected passages that address hunger, bread, cooking, taste, and smell, as they relate to the food-memory theories of David E. Sutton (2001) and Jon D. Holtzman (2006).

**Results:**

The results show that in La Grande Maison, food is an absence, a hunger, a poverty, a lack of bread that keeps alive the painful memory of colonial persecution and collective Algerian suffering. Food in Crescent, on the other hand, works by the presence of others: presence through shared tables and through diasporic memories and identities recreated with the Arab meal. In both novels, food serves as a shared historical experience that is remembered from one generation to the next, even as there are differences.

**Conclusions:**

The novelty of the paper is that it does not take into account the comparative aspect of food absence and presence within a single food-memory concept, which is not studied in the pair of novels. It extends the scope of comparative literary and food studies by enriching discourses on food, memory, and identity in colonial and diasporic writings. This multifaceted perspective can be applied to other fields of study, such as trauma, migration, and cultural studies in other postcolonial and diasporic settings, in future research.

## Introduction

In literature, food is used to symbolize, thus culturally, to signify identity, memory, belonging, exile, and trauma ( Sutton, 2001; Adel et al., 2025; Khater, 2023). The current critique has acknowledged cooking rituals, food, bread-making, and sensory experiences as a means to revive historical consciousness and transmit collective memory from one generation to the next ( Mihalache, 2026). Food, on the other hand, is deeply associated with displacement, homeland, and cultural survival in colonial and diasporic narratives ( Sparks, 2016; Nggilu et al., 2025; and Khater et al., 2025). In this context, Mohammed Dib’s La Grande Maison (1952) gestures towards food and images of hunger and poverty, as well as colonial deprivation, while Diana Abu-Jaber’s Crescent (2003) reminds us how cooking and hospitality help maintain a diasporic identity and how it can be used as a tool to re-engage with the homeland.

This study draws on the ideas of Sutton (2001) and Holtzman (2006) to examine how food is used in the two novels as a medium for memory and identity. Chosen for their unique and complementary historical experiences—colonial Algeria and the Arab American diaspora ( Adel et al., 2024)—the novels enable a comparison of food as a memory medium in material deprivation and cultural displacement. In both, food is used to describe memory and identity, but in opposite ways—absences are one thing, presence another.

While there is extensive scholarship on colonial deprivation and diaspora, food and cultural belonging have been explored separately and together by other scholars of *La Grande Maison* and *Crescent,* respectively, but not yet under a single framework of food-memory. Therefore, the absence and presence of food as complementary forms of memory formation have not been studied extensively. The colonial poverty, hunger and suffering of pre-independence Algeria are themes of scholarship on Dib’s novel with hunger and poverty as evidence of the French colonial exploitation in Sparks (2016), Omar’s child view reflecting the social realities of pre-independence Algeria as evidence of colonial pressure and collective consciousness in Woodhull (2000) and Omar’s persistent search for bread as evidence of collective identity in Alachaher Benzerdjeb (2016). But Guendouzi (2008) concludes that fear, paralysis, and scarcity rule the novel and permeate the novel’s characters at Dar-Sbitar. Furthermore, Guerid and Hammouda (2021) explain that Aïni’s kitchen is not her comfort zone, but a place where she was never able to fulfill her mission as a mother. The fact that these studies show that food absence in *La Grande Maison* is not just a minor detail but a reality that speaks to the violence of the colonies, social instability, and collective suffering, makes for an interesting reflection.

By contrast, criticism of Diana Abu-Jaber’s Crescent perceives food as a progressive force that reconstructs identity and belonging within the diaspora. Mehta (2009) argues that gatherings around Arab food in Nadia’s café in the novel create a substitute homeland. Through such sensory experience, Arab expatriates emotionally feel at home and culturally celebrate their identity even in diaspora. Similarly, Berrebbah (2020) claims that cooking rituals, recipes, and hospitality are strategies of encouraging intercultural communication among characters of different backgrounds. Further, Sena (2011) argues that food in *Crescent* carries emotional, historical, and political meanings related to exile, memory, and cultural belonging rather than functioning as a merely decorative culinary image. Collectively, critics agree that food in Abu-Jaber’s novel serves as a temporal carrier of memory, identity, communal belonging, and continuity for Arab immigrants in a displaced space.

Regardless of the value of these critical responses, most previous studies have treated the two novels separately and within distinct literary and cultural frameworks. Few studies compare how food functions in each novel oppositely yet interconnectedly, as absence and deprivation in *La Grande Maison* and as presence and abundance in *Crescent.* Hunger, nostalgia, exile, and identity have been the focus of previous readings, yet little work has been done on the comparative regard for food and memory in colonial and diasporic settings. In this paper, we will explore the connections and differences between the two novels and the role of food, which becomes more than sustenance: it is a medium that holds historical memory and creates collective identity.

The value of this study lies in its comparative food studies approach and engages with postcolonial literary studies and diasporic criticism ( Alhourani et al., 2025b; Rashwan, 2024; Rashwan, 2021) to consider the two novels under one analytical lens, despite their geographic and historical distinctiveness. The reading compares hunger and hospitality as two opposite facets of food memory, and of absence and presence as two opposing types of food as they are experienced in colonial and diasporic literature. In particular, it builds on current scholarship by theorizing the experience of food absence and presence as two forms of memory, shaping absence as colonial deprivation; presence as diasporic reconstruction, hospitality, and belonging. The paper begins with a theoretical explanation of food-memory studies and scholarship related to it, followed by an explanation of the methodology and a comparative analysis.

Accordingly, this study is guided by the following research question:

**RQ1:** How do Mohammed Dib’s *La Grande Maison* and Diana Abu-Jaber’s *Crescent* employ food as a medium of memory and identity formation, and how do food absence and food presence function as distinct yet interconnected responses to colonial and diasporic experiences?

**Figure 1.**
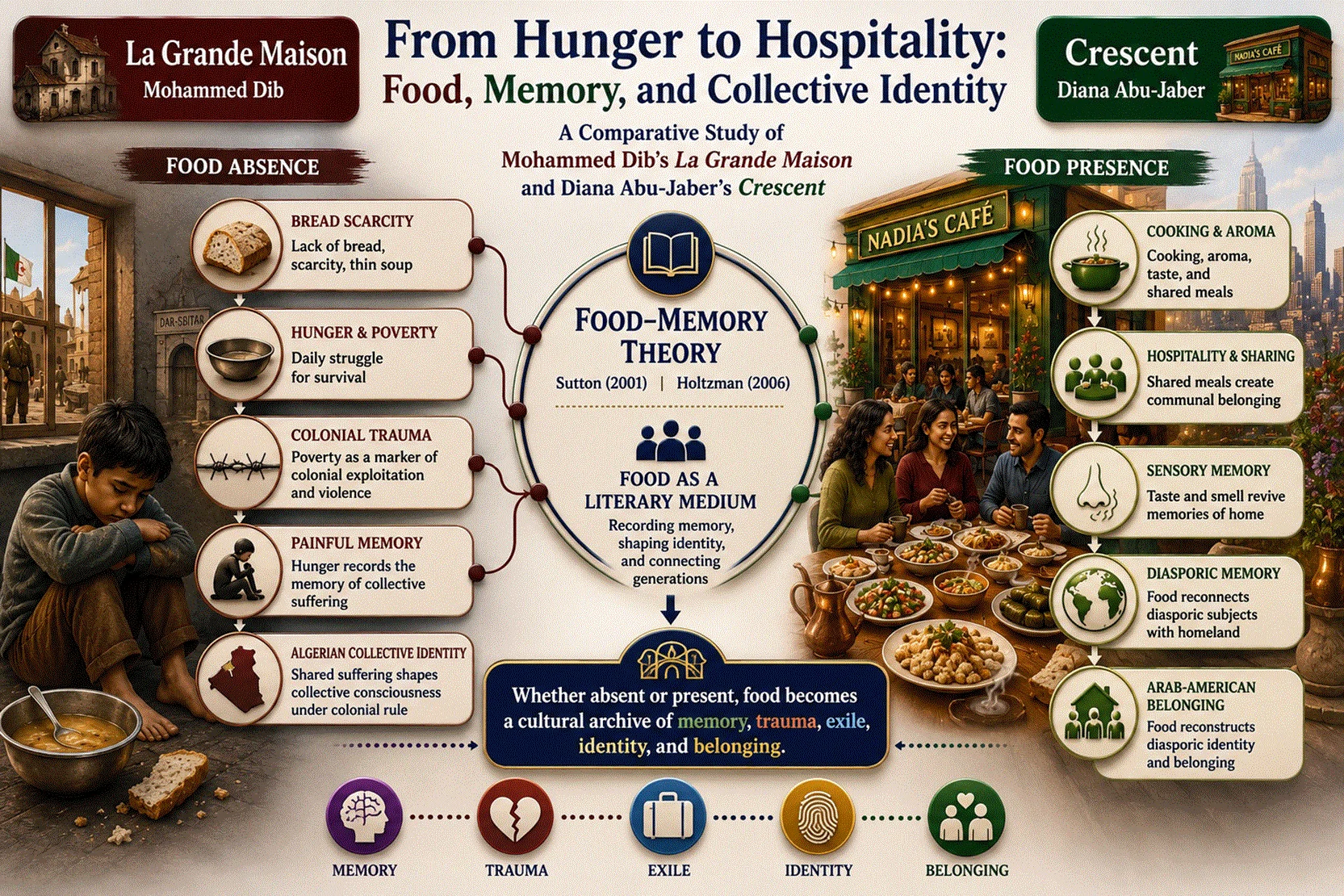
Food, Memory, and Collective Identity in *La Grande Maison* and *Crescent*: From Hunger to Hospitality. This figure shows the conceptual framework that is used to compare the study. Food is invoked in the absence of food in La Grande Maison, an emblem of the scarcity of bread, hunger, poverty, colonial oppression, and collective trauma that inform the collective memory and identity. Crescent is about food, and cooking, hospitality, sensory experience, and communal eating are key ways to maintain cultural identity and sense of belonging in diasporic communities. The figure illustrates the food-memory theory by Sutton (2001) and Holtzman (2006), which posits that both the scarcity and the surplus of food serve as mnemonic moments between memory, trauma, exile, identity, and belonging.

Figure 1 visually summarizes how food operates as absence in *La Grande Maison* and as presence in *Crescent* to clarify the study’s comparative logic, while linking both forms to memory, identity, trauma, exile, and belonging.

### Theoretical framework

David Sutton’s food-memory theory is a relevant scholarship that should be practical for this study. It considers food as more than an everyday material necessity. In *Remembrance of Repasts*, Sutton (2001) confirms that food is strongly related to culture, memory, embodiment, and material experience, especially in settings of migration and dislocation across borders. Then, Food can transmit personal and collective memory connotations. Food memory is intensely attached to the senses. Taste and smell are not neutral physical experiences; they often revive emotional memories and give meaning to the past. For example, a smell, a taste, a meal, or a humble recipe may transport the past into the present, allowing characters to remember people, places, and cultural origins ( Adel, 2023). This concept is significant to the present study because both *La Grande Maison* and *Crescent* use food to connect individual experience with wider histories of belonging, loss, and identity.

Recent food-memory scholarship settles that food memories are simultaneous to identity, nostalgia, trauma, place, and rituals ( Mihalache, 2026; Alhourani et al., 2025a). This is particularly and typically demonstrated in *Crescent*, wherein food rituals impel characters to remember their original Arab culture and recreate a sense of home in diaspora. In Abu-Jaber’s novel, food is not merely a source of nourishment for the restaurant clients. Instead, it functions as a motivating power to create a sensory bridge between the lost homeland and the present Arab American space.

Food rituals such as cooking and eating are also social performances that associate individuals with family, society, and homeland, ultimately creating a collective cultural identity ( Alenezi et al., 2026). Recent scholarship further demonstrates that food functions not only as a cultural and sensory medium but also as a symbolic and political language through which broader issues of identity, belonging, displacement, and social realities are articulated. In this regard, this study assumes that food is a communicative medium that connects memory and identity in colonial and diasporic settings, as Al-Khayyat and Abu Amrieh (2026) suggest, stating that food is a “sociopolitical marker of collective experiences and historical conditions.” According to Counihan and Van Esterik (2008), eating practices involve identity, belonging, resistance, and power and are shaped by culture and social relations. In the kitchen, table, and café, it becomes a space for collective memory and an emphasis on identity. Crescent proves itself to be a useful framework for reading the Arab food, hospitality, and café space as emotional and cultural connectors for the displaced characters.

However, food memory is not always produced through abundance or pleasure. Lack of food can also create strong memories, especially memories of agony, poverty, inequality, and colonial discrimination. Holtzman (2006) shows that food can represent ethnic identity, nationalism, exile, nostalgia, and sensual memory. In addition, he reports that hunger, lack, and poverty may be more reflexive than taste and smell in many communal and historical contexts. This can be an applicable perception to read *La Grande Maison*, in which hunger and rarity affect memories of Omar’s childhood and correspond to the suffering of the colonized Algerian society.

Therefore, this paper uses Holtzman’s and Sutton’s food-memory frameworks to study the depiction of food in two different forms. First, food as absence in *La Grande Maison* and second, food as presence in *Crescent.* In Dib’s novel, absence of food creates a painful memory of colonial hunger, poverty, and social oppression. In Abu-Jaber’s novel, presence of food refreshes memory through cooking, taste, and hospitality. According to this framework, food in the two novels, whether present or absent, becomes a literary tool for shaping identity, remembering history, and adapting to a substitute homeland.

Various literary studies have examined hunger, poverty, and colonial oppression in *La Grande Maison*, as well as diasporic belonging, nostalgia, and food in *Crescent.* Critics such as Guerid and Hammouda (2021), Sparks (2016), and Guendouzi (2008), focus on hunger, deprivation, and domestic struggle in Dib’s novel, while Aldemir (2025), Berrebbah (2020), and Mehta (2009) shed light on culinary memory, hospitality, and Arab American identity in Abu-Jaber’s work. Recently, Owaydhah (2023) examined Crescent as a postcolonial and diasporic text, suggesting that ethnic food serves as an indicator of cultural identity, memory, and belonging, thereby enabling diasporic subjects to maintain connections to their homeland and navigate their identities within the host culture. Most criticism, however, analyses the two novels separately, rather than employing a comparative food-memory framework, and ignores the interconnection of food-absence and food-presence as intertwined ways of maintaining historical memory and constructing collective identity. This study examines the two novels in order to highlight their differences and similarities in depicting food as something beyond nourishment.

## Methods

Using a qualitative comparative literary approach, this research investigates how food is represented in the novels by Mohammed Dib (La Grande Maison) and Diana Abu-Jaber (Crescent), and how the works make use of the concept of food-memory studies, especially Sutton (2001) and Holtzman (2006) who have conceptualized food as a sensory and cultural medium that carries memories, identities, nostalgias, and feelings of belonging. The analysis takes a comparative look at food in two juxtaposing but interdependent manifestations, absence in La Grande Maison and presence in Crescent.

A close textual analysis of quotes on hunger, bread, cooking, hospitality, taste, smell, and communal eating occasions is conducted, with greater focus on recurring or less satisfactory culinary images, domestic rituals, sensory narratives, and eating occasion settings. Food is not just read from the surface but also read for its figurative meanings, which carry forward historical awareness and help to create a collective identity. *La Grande Maison* is a work that centers on themes of hunger, poverty, and lack of bread as symptoms of colonialism and Algerian collective memory. Crescent’s focus is on Arab recipes, cooking, hospitality, and Nadia’s Café as cultural devices that enable diasporic subjects to reengage with the homeland and to reestablish their sense of belonging.

A comparative perspective highlights the similarities and differences in the two novels’ depiction of food and memory. The two novels take place in different historical and cultural contexts (colonial Algeria, the Arab American exodus), but both employ the language of food to construct and preserve memory and identity. By examining the various representations of food in its absence and presence, we gain deeper insights into how food represents trauma, helps maintain culture, and how it contributes to the production of a sense of belonging. This approach combines food memory and comparative literature theories to explore how food functions as a symbol of culture, identity, and emotion beyond its nutritional role.

## Results and discussion

### Food as absence in mohammed dib’s *La Grande Maison*

Sparks (2016) links food in *La Grande Maison* to scarcity and absence, while Woodhull (2000) identifies hunger as a force shaping identity and collective memory. The novel opens with Omar asking for food: “Un peu de ce que tu manges!” (p. 4). Such an opening is significant because Omar, as a child, is introduced through hunger rather than education, dreams, or family affection. His primary narrative position is that of a child whose body is already shaped by lack. In that way, hunger becomes the former verbal of identity in the novel: Omar recognizes himself and his surroundings through the continual experience of food shortage. Correspondingly, Alachaher Benzerdjeb (2016) notes that the central thread of this literary work is the search for nutrition, remarkably bread, and that Omar’s childhood is defined by rarity and deprivation.

Food scholarships such as by Holtzman (2006), Jones (2007), Strand (2023), and Sutton (2001) often treat food as a carrier of identity, memory, social value, and cultural meaning. That is why bread occupies a central role in the novel because it is both common and almost unreachable. One simulation of food as absence is the scene in which Aïni serves a watery soup without bread and her children hungrily kept asking about it. Sparks (2016) directly relates such scenes of missing bread to the colonial deprivation in Dib’s trilogy. Another example of food scarcity is the repeated sentence “le pain manquait” (p.30). While the sentence is short and simple, its repetition with the word “pain” makes bread more visible through its absence. For Jones (2007) and Mintz and Du Bois (2002), bread signifies dignity, security, and stability. Omar’s question, “Une tarechta sans pain?” (p. 28), reveals hunger as a cultural marker, while bread’s absence turns the family meal into a scene of tension and humiliation ( Sparks, 2016). And creates a deeply painful memory, as Strand (2023) conceptualizes. Through such scenes, Dib illustrates how colonialism is present in the very smallest domestic ritual to damage even the deepest emotional relations that between mother and children. Woodhull (2000) corresponds with this idea that shows Dar-Sbitar as a space marked by poverty, fear, and social pressure instead of being the warmest place that gathers the family. Also, Guerid & Hammouda (2021) and Jansen (1987) reconsider Aïni’s kitchen as a space of impossible responsibility instead of being the space of abundance and satiation.

As a mother, Aïni’s role was more than just cooking and serving; she tried to silence hunger, but she knew that she could not defeat it. The meal depiction as “une soupe de pâtes hachées et de légumes” already decreases its representation to a minimal act of survival rather than nourishment (p.28). Thus, the children swallow it immediately and instinctively, though they know it would not satisfy them. Whereas the hot soup gives momentary physical warmth, it also reawakens hunger rather than ending it. Even its spices are described as burning the children’s tongues, “leur cuisait la langue,”; it forces them to drink and drink again until they should be satisfied by drinking water, not by eating food (p.29). This representation extends beyond a simple depiction of poverty. Rather than alleviating suffering, food exposes it ( Strand, 2023). The irregular timing of meals becomes a daily reminder of deprivation and loss. While Sutton (2001) argues that food preserves memory through sensory experience, *La Grande Maison* constructs memory through absence: Omar remembers hunger, shame, and waiting rather than abundance. Similarly, Guendouzi (2008) describes Dib’s narrative as structured by hunger and fear, which drain the vitality of Dar-Sbitar.

Given that the families living there are impoverished, Dar-Sbitar exposes hunger as a group phenomenon, not an individual one. The meaning of the search for bread, neighborly assistance, and the hungry children in one family reflects pre-revolutionary Algeria as a whole ( Elimelekh, 2015). As the narrator explains, “A Dar-Sbitar, Omar se procurait du pain d’une autre façon,” and Yamina sometimes rewards him with “une tranche de pain” after small services (p. 5). The scene becomes more collective when Omar sees another hungry child secretly seize “un croûton” and bite into it, a moment that makes him cry because he recognizes hunger outside his own body (p.6). Woodhull notes that the novel presents events through Omar’s childhood perspective while connecting that viewpoint to the larger political and colonial circumstances of Algeria ( Woodhull, 2000). Gradually hunger becomes one of the early forms of political awakening. Primary, Omar feels injustice through his empty stomach before he can understand it politically. In other words, the writer connotatively embodies collective memory of colonial deprivation Omar’s hunger that throughout the novel plot becomes a first step toward social and national consciousness ( Desplanques, 1992; Elimelekh, 2015).

In *La Grande Maison*, arbitrariness of colonialism through police control and political anger on Algerian community is placed within scenes of domestic deprivation including empty food tables. For example, when Ben Sari condemns colonial “justice,” he refers to a system that fortifies the colonizer and convicts the colonized before any wrong is proven. Immediately after this political moment, the narrative returns to Aïni’s poor meal: “Aïni versa le contenu bouillant de la marmite,” but what she serves is only “une soupe de pâtes hachées et de légumes” (p. 28). Here, the shift is meaningful because it bridges political injustice expressed in Ben Sari’s declaration and the family’s failure to satisfy their body needs. Quickly, Dib adds: “Rien de plus, pas de pain” and Omar’s shocked question, “Une tarechta sans pain?” makes the absence of bread the key to the scene as it is fundamental emotionally as well as physically (p. 29). In brief, Dib relates colonial violence closely to the body. Oppression is not only experienced through police, prisons, or legal authorities, but also through the missing bread, the watery soup, and the mother’s failure to satisfy her children’s hunger. Accordingly, Sparks (2016) supports this reading by arguing that the novel gives actual image to colonial economics through hunger, expropriation, and the unequal distribution of food. Therefore, the movement from Ben Sari’s political anger to Aïni’s poor meal should not be accidental. It shows that colonial oppression becomes evident in the domestic habit of eating, where hunger turns political injustice into daily bodily pain.

Besides, the essence of the novel lies in its ability to make hunger a form of memory ( Sparks, 2016). The absence of food saves the memory of colonial poverty more effectively than a direct political speech would do ( Holtzman, 2006). Omar’s frail body, empty stomach, deprivation, and meager or deficient nutrition record colonial history in the novel. Education, family, entertainment, or pleasure might not be part of his childhood memories. Instead, his childhood is depicted through absence of bread, watery soup, waiting for food aid, shame, and fear. Even later in the novel, when public events become more noticeable, the demand for bread remains urgent. For example, Aïni’s question, “On ne mange pas parce que c’est la guerre?” reveals the correlation between historical events and everyday survival (p. 96). War may lead to political change, but for the poor, the immediate question remains whether there will be food on the table. This connects Dib’s political portrayal to realism since history is not abstract, but it is lived through hunger. Thus, bread, hunger, and poverty are not decorative motifs but the language of suffering and awakening. Dib shows colonialism destroys not only political freedom but also ordinary dignity, family peace, and childhood security. Through food-memory theory, the novel suggests absence can remember as deeply as presence: missing food becomes the very object through which a community’s identity and historical consciousness are preserved ( Holtzman, 2006).

### Food as presence in diana abu-jaber’s *Crescent*

In *Crescent*, food is a productive, positive force creating intimacy, memory, and cultural belonging. Unlike Dib, Abu-Jaber makes food visible, odorous, and socially active. Sirine works at Nadia’s Café, a Lebanese restaurant in Los Angeles that serves as a diasporic meeting place for Arab immigrants, exiles, students, and intellectuals. The menu offers “Real True Arab Food” (p. 21). Such a claim is significant because it denotes that food is not treated as ordinary consumption. It is presented as a nostalgic return to cultural origin. According to Sutton’s food-memory, this scene can be seen as an example of how food incorporates the past into the present, especially in migrant and transnational contexts where memory is often attached to taste, smell, and food practices ( Sutton, 2001). Therefore, the café becomes a space where Arab identity is, performed, remembered, and emotionally renewed through food gatherings.

For Sirine, personal and family memories are directly bonded to rituals of cooking. She returns to her parents’ old recipes and prepares the “almost forgotten” dishes of her childhood as soon as she begins working at Nadia’s Café. Emotionally, this practice turns her to her “parents’ tiny kitchen” and to her earliest memories (p. 19). Grounded to food-memory theory, this moment is central because memory is not exhibited as an abstract mental process. It is stimulated through the body senses of touching, smelling, seeing and tasting the ingredients. Sutton (2001) and Mihalache (2026) also stress that food transmits identity, ritual, knowledge, and everyday memory and is strongly related to embodiment and material culture. Thus, the act of cooking by Sirine becomes an embodied archive since she does not simply remember her Arab background mentally, yet she recovers it through recipes, ingredients, and culinary gestures.

In addition, identity in the novel is inherited through domestic practices of cooking that reveal sense of belonging. For Sirine, her parents were the original source of delicious recipes, especially her American mother, who is described as thinking about food “like an Arab” (p.39). Her Arab identity is transmitted not only through language, but also through dishes associated with her family such as labneh, mjeddrah, lamb, and stuffed grape leaves. In this case, it is significant because she is neither proficient in the Arabic language nor possessed of a stable sense of Arab identity. Food fills this gap. This kind of identity she accesses is sensory, intimate, and practical; basically, achieved through cooking. As Holtzman (2006) claims, food is deeply linked to identity, exile, and nostalgia, and it is the medium to make a collective memory in the text. Thus, food in *Crescent* serves as the agent through which Sirine celebrates her hybrid Iraqi-American self.

The substitute homeland is Nadia’s Café, wherein Arab students and immigrants gather to discuss common issues, create temporary connections with similar groups, and feel at home while eating old recipes. In such scenes, Abu-Jaber creates an emotional sense of belonging through their discussions and an actual identity through cooking, eating, and serving Arab food. She notes that for many of them the café offers “a little flavor of home” (P.19–20). This is one of the clearest applications of food-memory theory in the novel: taste becomes a way of recreating place. In Los Angeles, homeland does not actually exist; however, it is sensorially restored through recipes of bread, coffee, spices, and conversation. Analogously, Mehta (2009) insists that food in *Crescent* creates a diasporic space that associates characters across place and time and helps them reconnect with countries of origin. Thus, food transforms exile from a condition of loss and displacement into a shared cultural experience through which diasporic communities preserve memory, negotiate identity, and sustain connections with the homeland ( Owaydhah, 2023).

Another significant form of food-memory in *Crescent* is hospitality. The door of Nadia’s Café has always been open to welcome expatriates who are politically, nationally, and emotionally fragmented to join and enjoy Sirine’s cooking. They are Iraqi, Lebanese, Palestinian, Iranian, and other Middle Eastern characters who meet in the café, and food allows them to share a temporary sense of community. For example, Ramadan gatherings are especially important because people from different backgrounds gather around food such as killaj, qatayif, zalabiyya, and ma’mul (p. 297). This allows one to deduce that Arab identity in the novel is collective despite its diasporic sense. Difference does not disappear entirely; however, food creates a shared emotional space in which difference is held through coexistence. Berrebbah (2020) points out that the novelist uses culinary practice to structure Arab American identity and to create cross-cultural communication among characters of different nationalities and backgrounds.

In *Crescent,* Abu-Jaber teaches us the language of love and emotion that is shared between Sirine and Hanif through food. They connect through food, food preparation and sensory closeness. An Arab American woman who is looking for a richer sense of self in her father’s culture, and an Iraqi exile with a feeling of home in his memories of loss and political upheaval, are connected by a love of food. It lets Sirine connect with a part of her cultural memory that she never knew, and lets Hanif resurrect an ancient Arab world in the contemporary American scene. Mehta (2009) explains that the novel is full of smells: roasted lamb, rice, pine nuts, tabbouleh, knaffea—and not just smells of food, but also of “nostalgia, exile, identity, love, and reconciling with the past.” Aldemir (2025) is right, food in the novel isn’t just decoration; it has an emotional and historical significance.

However, Abu-Jaber does not entirely romanticize food. The novel acknowledges its temporality and instability. Um-Nadia’s statement that “Chefs know nothing lasts” (p. 57) reveals that food fades upon consumption, yet this very disappearance makes it memorable. This will further support Sutton's (2001) theory that food does not cause memory retention, but rather through its sensory aftertaste. Flavor has a fleeting quality, but it remains in the emotions. So, the novel is cooking as fragile, while also being powerful: a meal can vanish and yet can come back to bring back childhood, homeland, love, and culture.

In *Crescent*, food becomes presence, filling the café, collecting the exiled, bringing back the memory, binding the identity, and forming a sense of belonging. Cooking becomes a time and place of connection for Sirine’s past and present, her homeland and her diaspora, herself and her community ( Mehta, 2009). A Sutton’s food-memory reading of the novel shows that food can act as a sensory archive of Arab American identity ( Sutton, 2001). It reminds expatriates of migration, exile, loss, and political arbitrariness that have separated them. It uses cooking and hospitality to restore cultural memory and make belonging possible in the diaspora.

### Comparative discussion: From hunger to hospitality

Reading *La Grande Maison* and *Crescent* together exposes two contrasting but deeply connected literary uses of food. In the first work, food is memorized through missing bread, hunger, insufficient soup, deficiency, and the child’s anxious search for something to eat. In the second novel, food is remembered through presence: smell, taste, cooking, recipes, the café, and hospitality. Such comparison can be recognized through Sutton’s food-memory context, which identifies food as a medium of culture, embodiment, memory, and multinational belonging ( Sutton, 2001). Food as a code of the past through the body and senses is particularly significant in this context, as Sutton’s work documents, particularly in the context of migration and cultural dislocation. Food is not neutral in either novel; rather, a language of representation, a means by which the characters remember history, define identity, and claim belonging.

Memory operates differently in the two novels. In *La Grande Maison*, it is constructed through hunger and deprivation. Omar’s plea, “Un peu de ce que tu manges!” (p. 4), places childhood under the sign of scarcity, making hunger his first encounter with colonial inequality. Colonialism is experienced not as an abstract political system but as an empty stomach, anxiety in the kitchen, and the absence of bread at the table. The statement “pas de pain; le pain manquait” (p. 30) underscores how bread acquires meaning through its absence. Whereas Sutton (2001) argues that food is remembered through embodied sensory experience, Dib reverses this logic: the body also remembers what it cannot consume. Hunger thus transforms the body into an archive of colonial deprivation.

In *Crescent*, by contrast, memory is sustained through sensory presence. The café’s promise of “Real True Arab Food” (p. 21) turns food into a marker of cultural authenticity and a substitute homeland where displaced Arabs reconnect with their heritage. As Berrebbah (2020) notes, the novel enables Arab American expatriates to negotiate identity, belonging, and nostalgia through memory. Cooking, eating, smelling, and tasting become acts of remembrance, linking the American present to the Arab past.

The contrast is the key to the comparative argument. But in the novel, written by Dib, food memory is traumatic, defined by hunger, colonial inequality, and social suffering. Family dinners reveal the frailty of the family and bring a sense of humiliation and tension. In Abu-Jaber’s novel, however, food memory is restorative. Sirine’s meals create warmth and connection, offering immigrants and students “a little flavor of home” (p. 22). Taste not only recalls home but temporarily recreates it, allowing displaced characters to inhabit memory sensorially even when physical return is impossible.

Despite this difference, memory remains collective in both novels, recording the shared experiences of entire communities rather than isolated individuals. The hungry child in Dib’s novel symbolizes the general Algerians’ condition of suffering under colonization, in harsh living conditions, poverty, and lack. Likewise, food, cooking, and hospitality in Abu-Jaber’s novel are symbols and carriers of the collective memory and identity of Arab diasporic communities scattered from their homelands. Thus, whether built through absence or sensory presence, memory appears in both works as a communal archive of historical experience, cultural identity, and social belonging transmitted across generations.

Moreover, food also creates identity in distinctive yet parallel ways. In *La Grande Maison*, hunger reformulates Algerian collective identity under colonialism. The lack of food in Aïni’s house was never an individual daily issue, though it is personal. Dar-Sbitar is a distributed space of a poverty-stricken area where many families suffer similar conditions. Omar’s childhood, that is basically remarkable for a constant search for food, especially bread, reflects the larger suffering of the colonized Algerian community. Critics like Alachaher Benzerdjeb (2016) and Sparks (2016) note that *La Grande Maison* preserves the identity of colonial Algeria through a central thread of a constant process of food seeking. Therefore, hunger is a collective condition teaching Omar that poverty is not accidental but social, historical, and political.

On the contrary, in *Crescent*, food generosity makes Arab American diasporic identity. Sirine is not entirely associated with Arab identity through geography, language, or direct experience of homeland. Instead, she approaches the Arabic world through food, recipes, ingredients, cooking techniques, and the social rituals of feeding others. Abu-Jaber presents culinary practice as an essential cultural component through which diasporic figures identify themselves and declare affiliation with their homeland ( Berrebbah, 2020). Cooking is not just about inheritance, it is about Sirine’s identity. Arab food is the practice of an American hybrid identity when an American prepares an Arab dish in American society; it is not simply a repetition of the tradition.

Identity is shared in both novels, but is built differently in each. In the case of La Grande Maison, Algerian identity is defined by shared experiences of suffering, lack, poverty, and by an increased consciousness of prejudice. In Dar-Sbitar, poverty is a generator of crushing situations. In Crescent, Arab American identity is grounded in a communal generosity of the dispossessed in what they consume, how they communicate, and how they recall. Nadia’s Café demonstrates the power of diaspora to form a community of choice through food. Where one is sparse, one is more abundant, but both exhibit the production of identity as a collective and not a private process.

The two novels are separated by belonging. Dib’s work reflects a lack of dignity and justice amid hunger. Bread is a symbol of social value rather than nutritional value. In the absence of bread, the family will suffer not only from hunger but also lose their emotional balance and human dignity. Omar’s hunger is the experience of being discriminated against in the actual homes of the poor and even in the stomachs. As a result, La Grande Maison is considered a classic example of the Algerians’ fight against the French colonial regime, a record of poverty, hunger, discrimination, and the development of a collective struggle for liberation from a foreign power prior to independence. ( Elimelekh, 2015; Guendouzi, 2008). In this way, the novel becomes a form of political expertise, as hunger is its subject. It educates the poor that their sufferings are not just their own, nor have they existed in time immemorial, but that they are the result of history.

Hospitality and sharing meals make Crescent a place of belonging. In the café, for instance, there is a food table, where Arab immigrants, students, exiles, and intellectuals sit and temporarily reconnect, even though they are displaced from their homeland. In Berrebbah's (2020) description, the café is a diasporic space, and Abu-Jaber, through his culinary practice, calls for a sense of home. Food, in this place, is more than kindness, they’re a way of returning to culture. Sirine prepares meals for the displaced so they have a seat, something to talk about, to remember, and to be recognized. Omar’s world is defined by the question of whether there is enough bread, while Sirine’s world is identified by the question of whether food can gather fragmented histories and reshape a united community perspective.

The contrast between hunger and hospitality reshapes memory. In *La Grande Maison*, memory is tied to absence, poverty, colonialism, and suffering. In *Crescent*, despite exile and loss, memory is restorative, sustained through food, sensory experience, and communal meals. Food-memory scholarship links food to nostalgia, trauma, cultural revival, and the reconstruction of home ( Mihalache, 2026), making these novels suitable for comparison. Both use food to remember loss, but Dib emphasizes deprivation and awakening, whereas Abu-Jaber highlights belonging and renewal.

A close reading shows that the two novels demonstrate that food is a political sign. They reestablish the association between the individual body and shared history. Omar’s empty stomach reveals colonial injustice before political language can professionally exhibit it. Sirine’s cooking practice performs female Arab American identity before theory recognizes it. In both cases, the body becomes the place where memory and identity intersect. Here, Holtzman’s work on food and memory is useful because it relates food to sensual memory, nostalgia, ethnic identity, nationalism, exile, and social transformation ( Holtzman, 2006). Ultimately, Dib and Abu-Jaber dramatize these ideas in opposite directions: one through the wound of hunger, the other through the affective power of food.

So, the shift from hunger to hospitality is more than a comparison between two literary works. Rather, it is the basic comparative logic of the paper. *La Grande Maison* reveals that the absence of food can sustain memory of colonial anguish and reconstruct Algerian collective consciousness. However, *Crescent* establishes that it is the presence of food that can revive diasporic memory of homeland and create Arab-American collective belonging. In the first work, food memory is associated with material deprivation within the homeland, whereas in the second, it is associated with the emotional deprivation caused by separation from the homeland. In Dib’s work, food is powerful because it is missing, while in Abu-Jaber, food is powerful because it spreads among people. In *La Grande Maison*, the absence of bread gathers families to complain about their miserable present, however; in *Crescent* abundance of bread gathers people to complain about their past. Thus, the two novels show that food, whether absent or present, is one of the effective symbolic languages in literature to represent memory, identity, and the search for home.

## Conclusion

The study comparatively investigates the depiction of food in Dib’s *La Grande Maison* and Abu-Jaber’s *Crescent* through food-memory theoretical frame. It aims to explore how both novels convert food from a physical need into a literary medium that preserves memory and builds identity. Precisely, it addresses how food acts differently in each novel while keeping a shared connection with memory, belonging, and cultural experience. Through a comparative literary analysis based on the food-memory theories of Sutton (2001) and Holtzman (2006), the paper deduces that food in the two novels emerges from two opposite yet interconnected forms: absence in *La Grande Maison* and presence in *Crescent.*

The study finally concludes that food in Dib’s novel expands the realistic representation into a symbolic illustration of colonial violence and social injustice of the pre-independent Algeria. The permanent absence of bread and watery soup are material indicators of Algeria’s sufferance during colonialism. Lack structures the consciousness of Omar’s childhood and portrays the whole colonized community daily experience.

On the other side, food appears as productive and fertile component in *Crescent* through which memory is revived and diasporic identity is reshaped. Cooking, hospitality, and shared meals urged moved Arab characters to rejoin with homeland, preserve cultural continuity, and negotiate belonging within the American diaspora. Thus, while food memory in *La Grande Maison* is relevant to material deprivation within the homeland, food memory in *Crescent* reflects the emotional deprivation caused by dislocation from the homeland.

Despite these differences, the analysis also exhibits a significant convergence between the two works. In both novels, memory remains collective rather than purely personal since food records the cooperative experiences of entire societies. Whether stated through hunger or hospitality, food becomes a cultural archive through which identity, belonging, memory, trauma, and exile are inherited upon generations. Ultimately, the comparison reveals that food in literature exceeds its conventional role as source of nutrition and adopts political, emotional, and historical contribution.

The contribution of this paper lies in its combination of comparative analysis and food-memory theory in one unified paper. Comparatively, it explores the relationship between food absence and food presence as two interconnected motifs of remembering history and forming identity. In doing so, it goes beyond existing literary critical analyses on the symbolic and cultural statements of food in colonial and diasporic narratives.

The study limitations lie in the restricted focus on merely two novels and a qualitative textual analysis centered on food-memory theory. Further studies can widen the scope by analyzing other North African, Arab-American, or postcolonial literary narratives through multidisciplinary approaches merging food studies with gender, trauma, or migration studies. Comparative analyses, including autobiography or cinema bridged to food, may also further interpret the relationship between food, memory, and identity across cultures and historical contexts.

Eventually, the paper ends that food in both novels acts as one of memory’s most significant indicators in literature. Food can be a missing link in a person’s life, which is missing as a form of hunger, or it can be a connecting thread in a person’s life, which is present as a form of hospitality; food registers historical experience, preserves collective memory, and redefines culture. Together, *La Grande Maison* and *Crescent* show how food is a material substance, but also a medium for literature, a testimony to human poverty, suffering, and hunger, and a means of holding onto identity and memory.

## Ethical considerations

N/A (the study involved no human participants, animals, personal data, or identifiable information).

## Data Availability

No datasets were generated or analyzed for this article. The study is a qualitative comparative literary analysis based on published literary texts and secondary scholarly sources; therefore, no underlying research data are associated with this article. No specific reporting guideline applies to this study because it is a qualitative comparative literary analysis based on published literary texts and secondary scholarly sources.
