# Supplementary material for: From Hunger to Hospitality: Food, Memory, and Collective Identity in Mohammed Dib’s La Grande Maison and Diana Abu-Jaber’s Crescent
Source: F1000Res. 2026 Sep 7;15:1487. [Version 1] doi: 10.12688/f1000research.184574.1 (PMC13612995; doi:10.12688/f1000research.184574.1)
Supplement: Peer review history

# Reviewer report: Reviewer response for version 1

**DOI:** 10.5256/f1000research.203732.r520336

**Published:** 2026-09-25

**Authors:** Emad Ali Alawad

**Affiliations:** 1 Modern College of Business and Science, Al Khuwair, Muscat Governorate, Oman

Summary

 The article From Hunger to Hospitality: Food, Memory and Identity in Mohammed Dib’s La Grande Maison and Diana Abu-Jaber’s Crescent analyses food as element of absence (La Grande Maison) or presence (Crescent) within the formation of identity, memory or belonging - using food-memory theory (Sutton 2001; Holtzman 2006). The comparison takes place along lines of colonial Algeria versus Arab-American diaspora experiences.

 Evaluation

 1. Statistical analysis: No source data required - qualitative study with interpretive framework only

 2. Source data availability: No source data required - texts are published and accessible. Authors should include passages analysed or coding framework.

 3. Conclusions supported: Yes - in line with text and theory. Slight improvement: make analytical steps more explicit.

 4. Clarity and literature: Partly - presentation clear but citations of somewhat older sources. Include more recent work on food, identity and diaspora/postcolonial studies.

 5. Study design: Yes - qualitative comparative design appropriate and sound

 6. Methods detail: Partly - lacks criteria for choosing passages or categories for coding and balancing analysis. Such inclusion would enhance study rigor.

 Required Revisions: 

 1. Expand literature review with recent scholarship

 2. Clarify methodological procedures - selection criteria, coding categories and steps

 3. Document analysed material (appendix of excerpts or coding scheme)

 Overall Recommendation

 The study is strong conceptually and well-designed - but requires methodological clarification and updated literature engagement to become scientifically sound.

**Recommendation: Revisions required before acceptance.**

Is the work clearly and accurately presented and does it cite the current literature?

Partly

If applicable, is the statistical analysis and its interpretation appropriate?

Not applicable

Are all the source data underlying the results available to ensure full reproducibility?

No source data required

Is the study design appropriate and is the work technically sound?

Yes

Are the conclusions drawn adequately supported by the results?

Yes

Are sufficient details of methods and analysis provided to allow replication by others?

Partly

Reviewer Expertise:

Corpus Linguistics, Bilingual Education, Business Communication, Academic Writing, Curriculum Design, Applied Linguistics and Language TeachingI also have considerable experience in teaching design and learning analysis, assessment analysis and pedagogical alignment with a specific focus on cultural and/or linguistic awareness in language teaching, referencing and research writing skills.

I confirm that I have read this submission and believe that I have an appropriate level of expertise to confirm that it is of an acceptable scientific standard.

# Reviewer report: Reviewer response for version 1

**DOI:** 10.5256/f1000research.203732.r520338

**Published:** 2026-09-24

**Authors:** Faiz Algobaei

**Affiliations:** 1 Al-Fayha Private College, Al Jubail City, Saudi Arabia

The manuscript presents an interesting and well-focused comparative analysis of food, memory, and collective identity in La Grande Maison and Crescent. The contrast between food as absence and food as presence provides a clear perspective and contributes meaningfully to the discussion of food and memory in colonial and diasporic literature. However, a few minor revisions would further improve the manuscript:

 1. The manuscript would benefit from careful proofreading to correct minor grammatical errors and awkward expressions, particularly in the Results and Discussion section.

 2. Please carefully check the consistency of citations and references throughout the manuscript. For example, Sena is cited as 2016 in the text but listed as 2011 in the References, and “Holtsman (2006)” should be corrected to “Holtzman (2006).”

 3. In the abstract. (The novelty of the paper is that it does not take into account the comparative aspect of food absence and presence within a single food-memory concept, which is not studied in the pair of novels. It extends the scope of comparative literary and food studies by enriching discourses on food, memory, and identity in colonial and diasporic writings.) It appears to contain a serious wording error.

Is the work clearly and accurately presented and does it cite the current literature?

Yes

If applicable, is the statistical analysis and its interpretation appropriate?

Not applicable

Are all the source data underlying the results available to ensure full reproducibility?

Yes

Is the study design appropriate and is the work technically sound?

Yes

Are the conclusions drawn adequately supported by the results?

Yes

Are sufficient details of methods and analysis provided to allow replication by others?

Yes

Reviewer Expertise:

English Literature, Applied Linguistics, Translation Studies, and Artificial Intelligence in Language and Translation.

I confirm that I have read this submission and believe that I have an appropriate level of expertise to confirm that it is of an acceptable scientific standard.

# Reviewer report: Reviewer response for version 1

**DOI:** 10.5256/f1000research.203732.r520334

**Published:** 2026-09-21

**Authors:** Baker Bani-Khair

**Affiliations:** 1 The Hashemite University, Az-Zarqa, Zarqa Governorate, Jordan

The paper titled "From Hunger to Hospitality: Food, Memory, and Collective Identity in Mohammed Dib’s *La Grande Maison* and Diana Abu-Jaber’s *Crescent*" is highly suggestive and informative with a special focus on Food Memory and Cultural Identity Studies, using a suitable critical approach by both Sutton and Holtzman,  which significantly enriches the study. The author has successfully used the theory along with a detailed analysis that highlights the theme of food as a symbolic representation of suffering and exile within a postcolonial perspective in Mohammed Dib’s *La Grande Maison* and Diana Abu-Jaber’s *Crescent* .  The author's writing style and language are excellent with a lot of scholarly citations. I strongly recommend accepting the paper in your journal. I suggest consulting other more interesting papers that might enhance the author's paper if possible, but this is not a necessary request. Here are some scholarly papers that add additional insight to the theme of postcolonial dilemma of food memory, race, class, and gender:

1. Refer (1)
2. Refer (2)

Is the work clearly and accurately presented and does it cite the current literature?

Yes

If applicable, is the statistical analysis and its interpretation appropriate?

Not applicable

Are all the source data underlying the results available to ensure full reproducibility?

Yes

Is the study design appropriate and is the work technically sound?

Yes

Are the conclusions drawn adequately supported by the results?

Yes

Are sufficient details of methods and analysis provided to allow replication by others?

Yes

Reviewer Expertise:

Gothic literature

I confirm that I have read this submission and believe that I have an appropriate level of expertise to confirm that it is of an acceptable scientific standard.

## References

[1] Laboudi, H. (2024). Re-mapping the Postcolonial Heritage of Exile in Abdulrazak Gurnah’s Admiring Silence. Jordan Journal of Modern Languages & Literatures, 16(2), 543–555. https://doi.org/10.47012/jjmll.16.2.15.

[2] Ouatat, A. (2024). Discussing Exile and Diaspora through Food:Diana Abu Jaber’s Crescent, Journal of English Literature and Cultural Studies, 5 (3): 53-61.
